# Tumor-infiltrating lymphocytes and macrophages as a significant prognostic factor in biliary tract cancer

**DOI:** 10.1371/journal.pone.0280348

**Published:** 2023-01-24

**Authors:** Ryota Tanaka, Shimpei Eguchi, Kenjiro Kimura, Go Ohira, Shogo Tanaka, Ryosuke Amano, Hiroaki Tanaka, Masakazu Yashiro, Masaichi Ohira, Shoji Kubo

**Affiliations:** 1 Department of Hepato-Biliary-Pancreatic Surgery, Osaka City University Graduate School of Medicine, Osaka, Japan; 2 Department of Gastroenterological Surgery, Osaka City University Graduate School of Medicine, Osaka, Japan; 3 Department of Medical Oncology, Sidney Kimmel Cancer Center, Thomas Jefferson University, Philadelphia, PA, United States of America; 4 Molecular Oncology and Therapeutics, Osaka City University Graduate School of Medicine, Osaka, Japan; 5 Cancer Center for Translational Research, Osaka City University Graduate School of Medicine, Osaka, Japan; Texas A&M University, UNITED STATES

## Abstract

**Background:**

The impact of tumor-infiltrating lymphocytes (TILs) and tumor-associated macrophages (TAMs) on the prognosis of biliary tract cancer (BTC) is not completely understood. Therefore, in our study, we investigated the effects of the various immune cells infiltration in tumor microenvironment (TME).

**Methods:**

A total of 130 patients with BTC who underwent surgical treatment at our institution were enrolled in this study. We retrospectively evaluated TILs and TAMs with immunohistochemical staining.

**Results:**

With CD8-high, CD4-high, FOXP3-high, and CD68-low in TME as one factor, we calculated Immunoscore according to the number of factors. The high Immunoscore group showed significantly superior overall survival (OS) and recurrence-free survival (RFS) than the low Immunoscore group (median OS, 60.8 vs. 26.4 months, *p* = 0.001; median RFS not reached vs. 17.2 months, *p* < 0.001). Also, high Immunoscore was an independent good prognostic factor for OS and RFS (hazards ratio 2.05 and 2.41 and *p* = 0.01 and *p* = 0.001, respectively).

**Conclusions:**

High Immunoscore group had significantly superior OS and RFS and was an independent good prognostic factor for OS and RFS.

## Introduction

The tumor microenvironment (TME) is composed of not only cancer cells but also an extracellular matrix and many types of non-cancerous cells, including fibroblasts, myeloid cells, and lymphocytes [1]. Immune cells, such as lymphocytes, neutrophils, monocytes, and dendritic cells, found in TME are called tumor-infiltrating immune cells (TIICs) [2]. Among TIICs, our institute has reported that tumor-infiltrating lymphocytes (TILs) and tumor-associated macrophages (TAMs) are involved in tumor progression and serve as prognostic factors in colorectal and breast cancer [3–5]. In recent years, TIICs are also known as a predictor of chemotherapy and immunotherapy effectiveness [6, 7].

Although TILs play a central role in anti-tumor immune response, in recent years, TAMs have become known as an important factor involved in tumor progression [8]. However, Zhang et al. revealed that high-infiltration of TAMs is associated with unfavorable prognosis in patients with gastric, urogenital, and head and neck cancer while it is associated with favorable prognosis in patients with colorectal cancer [9]. Their correlation with cancer prognosis remains unclear.

Biliary tract cancer (BTC) has an unfavorable prognosis. In recent years, not only surgical treatment and chemotherapy but also immunotherapy has been developed. However, these treatments for BTC are not satisfactory. Currently, immune checkpoint inhibitors have a confirmed efficacy for patients with BTC [10]. Although many researchers have reported TILs and TAMs as prognostic factors in BTC [11–16], we believe that it is necessary to consider TILs and TAMs together as cancer immunity in TME. The purpose of this study was to evaluate infiltration with lymphocytes and macrophages in BTC specimens that have undergone surgery in our department.

## Materials and methods

### Patient and tissue samples

Clinical data and formalin-fixed paraffin embedded (FFPE) tissues were obtained from 130 patients who underwent surgical treatment for BTC at our institution between 2001 and 2017 (Table 1). BTC includes intrahepatic, perihilar, and distal bile duct cancer, gallbladder cancer, and ampullary cancer. The surgical treatment for intrahepatic and perihilar cancer comprised partial hepatectomy and major hepatectomy with or without bile duct reconstruction. Gallbladder resection and extrahepatic bile duct resection with or without regional lymph nodes dissection were performed for gallbladder cancer. Pancreaticoduodenectomy was performed for distal bile duct cancer and ampullary cancer. None of the patients underwent preoperative radiotherapy or chemotherapy. Pathological findings were retrospectively evaluated following the Japanese classification of biliary tract cancers, third edition [17]. The TMN classification was reclassified following the American Joint Committee on Cancer system, eighth edition [18]. After surgery, the patients were followed up at every 3- to 6-month with tumor markers and enhanced computed tomography until 60 months. Recurrence-free survival (RFS) and overall survival (OS) are defined as the time from surgery to cancer recurrence or death. This study conforms to the Declaration of Helsinki and was approved by the Osaka City University Ethics Committee (approval number 924). Written informed consent was obtained from each patient.

**Table 1:** Clinicopathological characteristics of 130 patients with BTC.

		number
Sex	men	71
	women	59
Age, median (range)		68.5 (43–87)
Location of cancer	peripheral and distal bile duct	56
	intrahepatic bile duct	20
	gallbladder	23
	ampullary	31
T category	pT0	15
	pT1	20
	pT2	41
	pT3	48
	pT4	6
Lymph node metastasis	absent	88
	present	42
Distant metastasis	absent	121
	present	9
Lymphatic invasion	absent	51
	present	52
Vascular invasion	absent	86
	present	17
UICC stage	0	15
	1	22
	2	57
	3	34
	4	2
Serum CEA level, ng/ml, median (range)		2.45 (0–86.5)
Serum CA19-9 level, U/ml, median (range)		30 (0–45152)
Chemotherapy	yes	71
	no	59
Recurrence	yes	65
	no	65
Outcome	death	62
	alive	68
Recurrence free survival, days, median (range)		544 (0–4160)
Overall survival, days, median (range)		786 (35–4157)
CD8 TILs, median (range)		40 (0–216)
CD4 TILs, median (range)		79 (0–330)
FOXP3 TILs, median (range)		21 (0–160)
CD68 TAMs, median (range)		92 (12–300)

BTC: biliary tract cancer, UICC; Union for International Cancer Control, CEA; carcinoembryonic antigen, CA19-9; carbohydrate antigen 19–9, TILs; tumor infiltrating lymphocytes, TAMs; tumor associated macrophages.

### Tissue microarray construction

Tissue microarray (TMA) blocks with one 3.0-mm-diameter punch core per tumor were constructed from FFPE tissue blocks of resected specimens from primary site, as previously reported [19]. We ensured that representative tumor cell-rich areas are H&E-stained with a light microscope and were sent to create TMA blocks (S1 Fig).

### Immunohistochemical staining

TILs and TAMs were examined by immunohistochemical staining using BX50 DIC microscope (Olympus, Tokyo, JP). CD68 antibody was used as a pan-macrophage marker. Sections with a thickness of 4 μm were obtained from TMA blocks. Immunohistochemistry was done as previously described [20]. Primary specific antibodies for CD4 (1:80 dilution; Dako, Glostrup, Denmark), CD8 (1:100 dilution; Dako, Glostrup, Denmark), FOXP3 (1:100 dilution; Abcam, Cambrige, UK), CD3 (1:100 dilution; Dako, Glostrup, Denmark), and CD68 (1:100 dilution; Leica Biosystems, Newcastle Upon Tyne, UK) were used.

### Evaluation of immunohistochemical staining

The immunohistochemical evaluation was performed by researchers independently. The number of TILs and TAMs around the tumor cells was evaluated with a microscope in three randomly selected fields at a magnification of × 400 and the average number was calculated (S2 Fig). All specimens were evaluated without any previous knowledge of the patients’ clinical background.

### Determination of cutoff values

**Fig 1:**
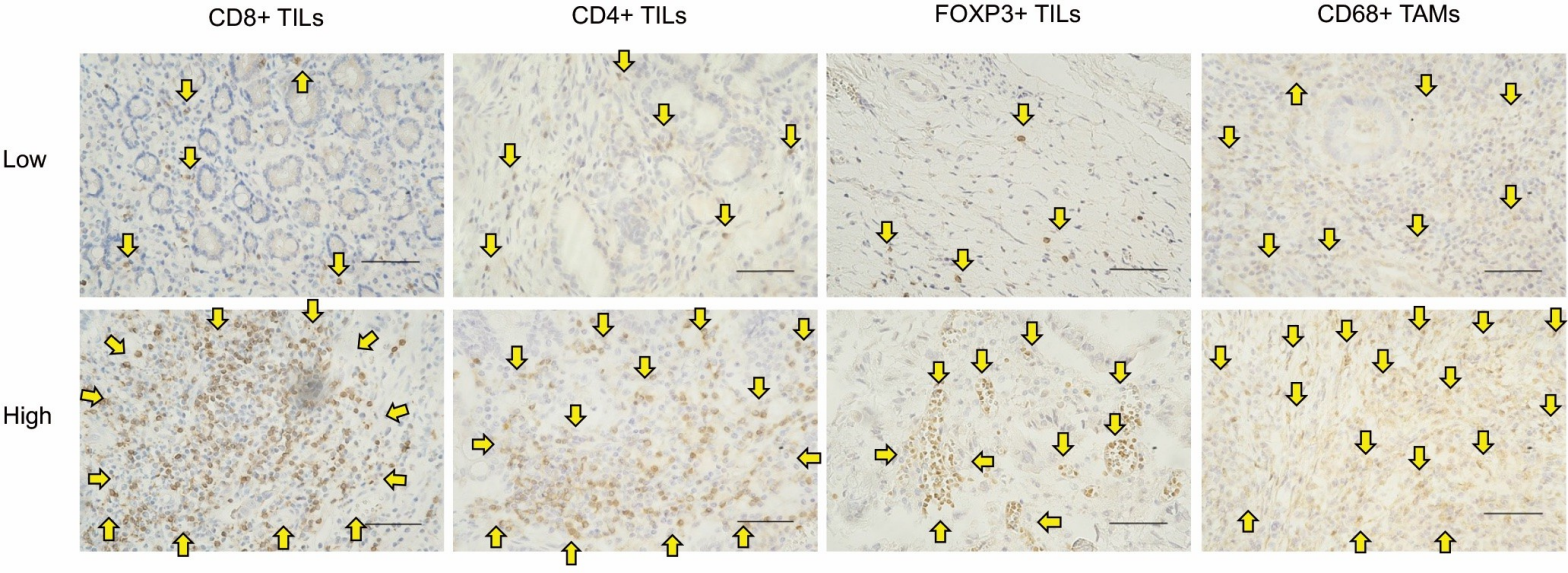
Immunohistochemical staining. Microscopic images showing high- and low-infiltration groups with CD8+, CD4+, FOXP3+, and CD68+ cells. Magnification is 400x, and the scale bar is 25 μm. Immune cells around the tumor are stained brown (arrows). Each patient is classified into the high- or low-infiltration group based on the cutoff value.

To set the cutoff values for the number of CD8+, CD4+, FOXP3+, and CD68+ cells, receiver operating characteristic (ROC) curve analyses for 5-year RFS were performed (S3 Fig). All patients were assigned into two groups, high-infiltration and low-infiltration groups, based on these cutoff values. The cutoff values were 40 for CD8+ cells, 48 for CD4+ cells, 29 for FOXP3+ cells, and 127 for CD68+ cells (Fig 1).

### Immunoscore in the tumor microenvironment

With CD8-high, CD4-high, and FOXP3-high TILs and CD68-low TAMs as one factor, the number of factors was counted in each case, and Immunoscore was assigned a score of 5 stages, from 0 to 4. Immunoscore was high for the score of 3–4 and low for the score of 0–2.

### Statistical analysis

Continuous variables were compared using the Mann-Whitney U-test. Categorical variables were compared using the chi-square or Fisher exact tests, as appropriate. Cox proportional hazard regression analyses were performed to identify prognostic predictors. The OS and RFS rates were estimated by the Kaplan–Meier method, and survival curves were compared using the log-rank test. Univariate and multivariate analyses were performed by cox regression hazard model. Groups were considered to be significantly different at *p* < 0.05. All tests were performed using JMP software.

## Results

### Evaluation of infiltrating immune cells and clinicopathological characteristics

Cases with the number of infiltrating CD8+ TILs that varied from 0 to 216/high power field (HPF) (median 40) (S2 Fig) and 55.4% (72 out of 130 cases) were assigned to the high-infiltration CD8+ TILs group. Cases with the number of infiltrating CD4+ TILs that varied from 0 to 330/HPF (median 79) and 69.2% (90 out of 130 cases) were assigned to the high-infiltration CD4+ TILs group. Cases with the number of infiltrating FOXP3+ TILs that varied from 0 to 160/HPF (median 21) and 39.2% (51 out of 130 cases) were assigned to the high-infiltration FOXP3+ TILs group. Cases with the number of infiltrating CD68+ TAMs that varied from 12 to 300/HPF (median 92) and 70% (91 out of 130 cases) were assigned to the low-infiltration CD68+ TAMs group. (Table 1). High CD8+ and CD4+ TILs were statistically significantly associated with gender (*p =* 0.04 and *p* = 0.009, respectively; Tables 2 and 3).

**Table 2:** Correlation between clinicopathological features and TILs in 130 patients with BTC.

		CD8 TILs		P value	CD4 TILs		P value	FOXP3 TILs		P value
		High	Low		High	Low		High	Low	
		N = 72	N = 58		N = 90	N = 40		N = 51	N = 79	
Sex	men	45	26	*0.04	56	15	*0.009	28	43	0.96
	women	27	32		34	25		23	36	
Age, median (range)		70.5(51–87)	67(43–86)	0.43	69(43–83)	68(43–87)	0.91	67(43–84)	69(46–87)	0.19
T category	pT0-2	40	36	0.45	53	23	0.88	33	43	0.24
	pT3-4	32	22		37	17		18	36	
Lymph node metastasis	absent	51	37	0.39	64	24	0.21	36	52	0.57
	present	21	21		26	16		15	27	
Distant metastasis	absent	67	54	0.99	85	36	0.35	49	72	0.28
	present	5	4		5	4		2	7	
Lymphatic invasion	absent	32	19	0.6	39	12	0.3	23	28	0.62
	present	30	22		35	17		21	31	
Vascular invasion	absent	54	32	0.23	63	23	0.47	36	50	0.69
	present	8	9		11	6		8	9	
UICC stage	≦2	54	40	0.44	68	26	0.21	40	54	0.21
	>2	18	18		22	14		11	25	
Serum CEA level	<5 ng/ml	61	40	0.14	76	25	0.12	43	58	0.97
	≧5 ng/ml	8	11		11	8		8	11	
Serum CA19-9 level	<37 U/ml	37	29	0.74	49	17	0.43	27	39	0.82
	≧37 U/ml	33	23		38	18		24	32	

*p < 0.05

TILs; tumor infiltrating lymphocytes, BTC; biliary tract cancers, UICC; Union for International Cancer Control, CEA; carcinoembryonic antigen, CA19-9; carbohydrate antigen 19–9.

**Table 3:** Correlation between clinicopathological features and TAMs in 130 patients with BTC.

		High	Low	
		N = 39	N = 91	
Sex	men	25	46	0.15
	women	14	45	
Age, median (range)		72(43–83)	67(43–87)	0.19
T category	pT0-2	19	57	0.14
	pT3-4	20	34	
Lymph node metastasis	absent	24	64	0.32
	present	15	27	
Distant metastasis	absent	36	85	0.82
	present	3	6	
Lymphatic invasion	absent	13	38	0.11
	present	21	31	
Vascular invasion	absent	29	65	0.73
	present	10	26	
UICC stage	≦2	29	65	0.73
	>2	10	26	
Serum CEA level	<5 ng/ml	31	70	0.59
	≧5 ng/ml	7	12	
Serum CA19-9 level	<37 U/ml	16	50	0.07
	≧37 U/ml	22	34	

TAMs; tumor associated macrophages, BTC: biliary tract cancers, UICC; Union for International Cancer Control, CEA; carcinoembryonic antigen, CA19-9; carbohydrate antigen 19–9.

### Association between TILs and survival outcomes

**Fig 2:**
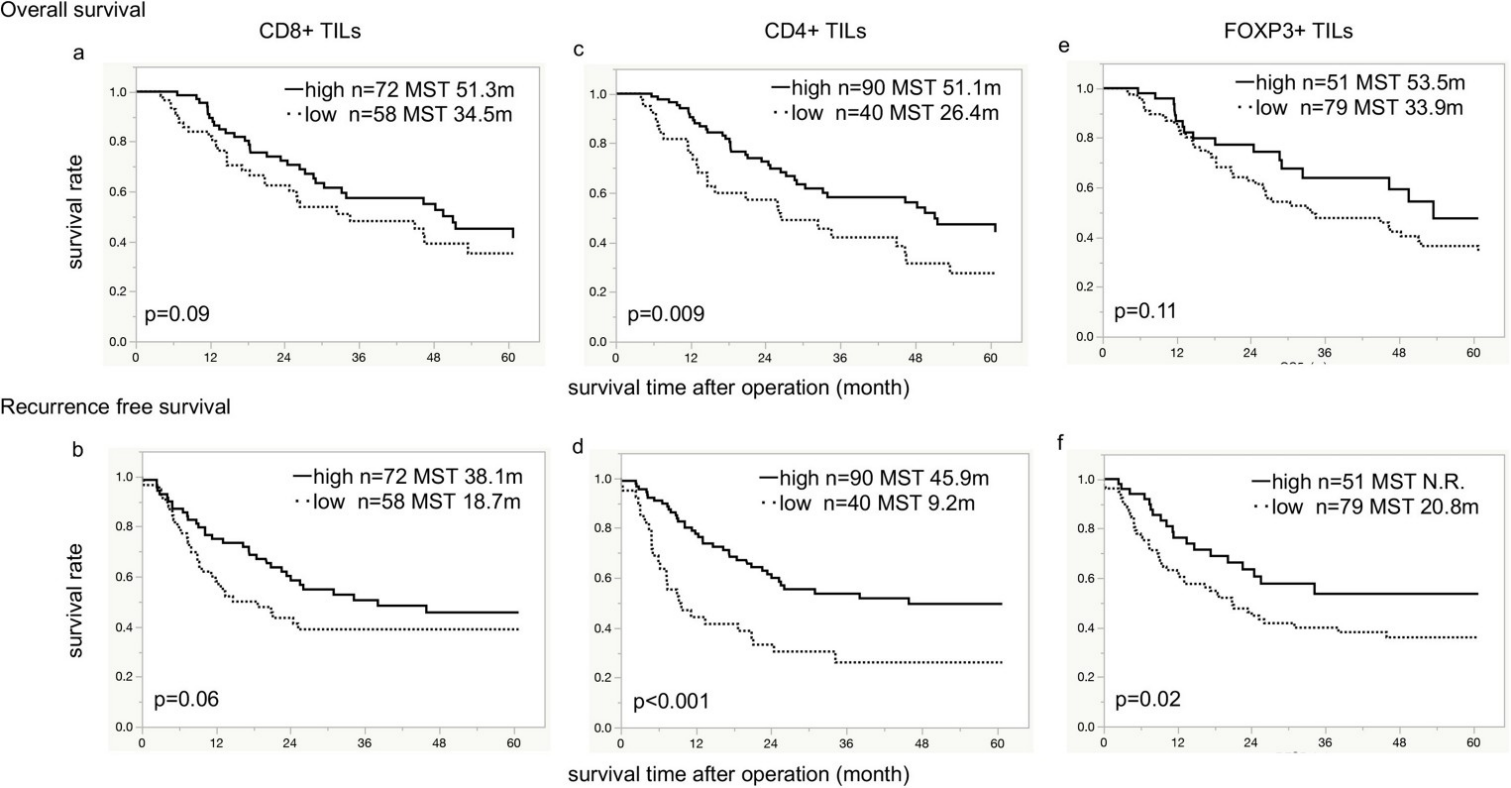
Overall survival and recurrence-free survival for tumor-infiltrating T cells. Kaplan-Meier survival curve indicates that the high-infiltrating CD4+ TILs group shows significantly superior OS and RFS than the low-infiltrating CD4+ TILs group. The high-infiltrating FOXP3+ TILs group shows significantly superior RFS than the low-infiltrating FOXP3+ TILs group. TILs: tumor-infiltrating lymphocytes, OS: overall survival, RFS: recurrence-free survival, MST: median survival time, N.R.: not reached.

In the entire 154-patient population, the high-infiltration CD8+ TILs group showed longer OS [20]. Due to the difference of the population in this study, the high-infiltration CD8+ TILs group showed the tendency of superior OS (median OS 51.3 months) compared to the low-infiltration CD8+ TILs group (median OS 34.5; *p = 0*.*09*; Fig 2A). Similarly, the high-infiltration CD8+ TILs group showed the tendency of superior RFS (median RFS 38.1 months) compared to the low-infiltration CD8+ TILs group (median RFS 18.7 months; *p = 0*.*06*; Fig 2B). Patients with high CD4+ TILs infiltration showed significantly superior OS and RFS than those with low CD4+ TILs (median OS 51.4 vs. 26.4 months, respectively, *p = 0*.*009*; median RFS 45.9 vs. 9.2 months, respectively, *p < 0*.*001*; Fig 2C and 2D). There is no significant difference in OS between patients with high FOXP3+ TILs infiltration (median OS 53.5 months) and those with low FOXP3+ TILs infiltration (median OS 33.9 months;*p = 0*.*11*; Fig 2E). Patients with high FOXP3+ TILs infiltration showed significantly superior RFS (median RFS not reached) compared to those with low FOXP3+ TILs infiltration (median RFS 20.8 months; *p = 0*.*02*; Fig 2F).

### Association between TAMs and survival outcome

**Fig 3:**
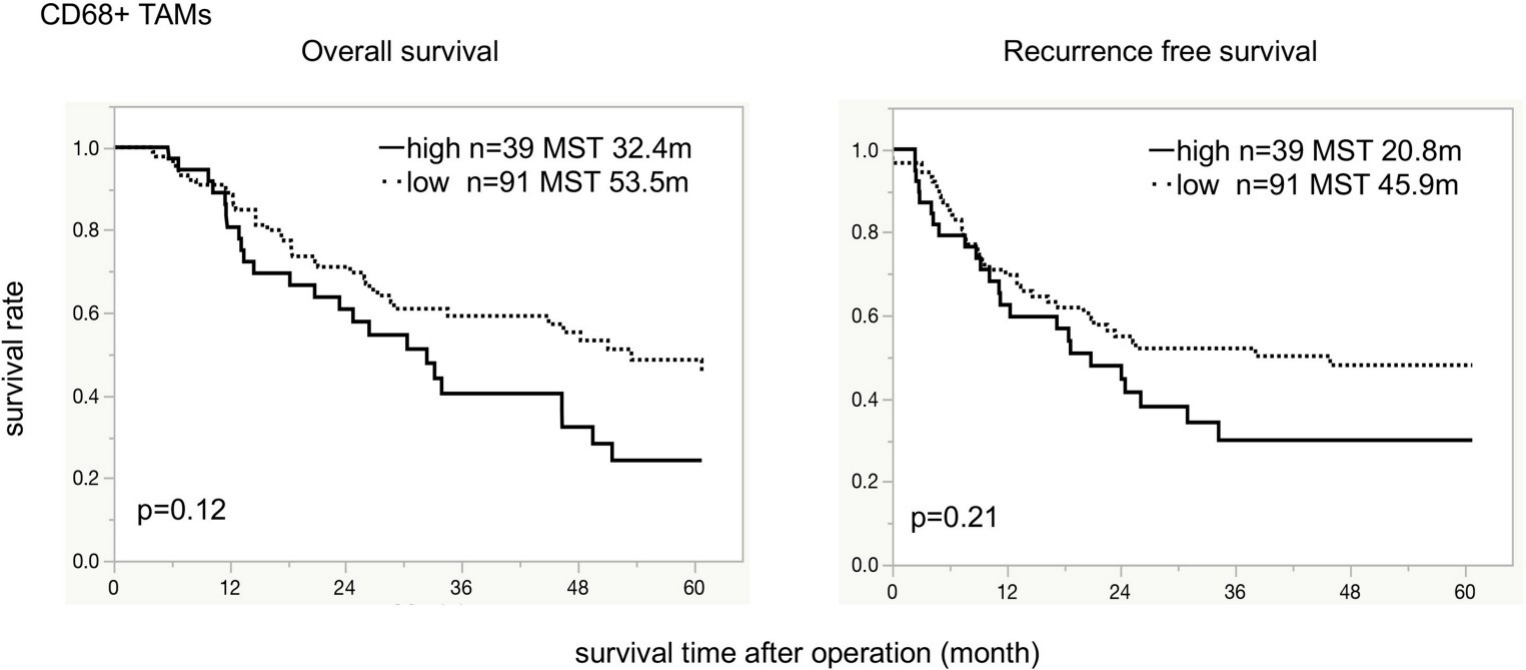
Overall survival and recurrence-free survival for tumor-associated macrophages. Kaplan-Meier survival curve indicates that there is no significant difference in OS and RFS between the high- and the low-infiltrating CD68+ TAMs groups. TAMs: tumor-associated macrophages, OS: overall survival, RFS: recurrence-free survival, MST: median survival time.

In the total patient population, patients in the low-infiltration CD68+ TAMs group tended to have superior OS (median OS 53.5 months) compared to the high-infiltration CD68+ TAMs group (median OS 32.4 months; *p = 0*.*12*; Fig 3A). Similarly, patients with low CD68+ TAMs infiltration showed the tendency of superior RFS (median RFS: 45.9 months) compared to patients with high CD68+ TAMs infiltration (median RFS 20.8 months; *p = 0*.*21*; Fig 3B).

### Univariate and multivariate analyses of TILs and TAMs

Univariate and multivariate analyses of the patients were performed with clinicopathological predictors and infiltrating immune cells for OS and RFS using the cox regression model (Tables 4 and 5). For OS, positive lymph node metastasis, presence of distant metastasis, and high serum CA19-9 level were independent poor prognostic factors (hazards ratio 2.0, 3.34, and 17.2, respectively; *p = 0*.*02*, *p = 0*.*007*, and *p = 0*.*04*, respectively; Table 4). For RFS, T classification (pT3-4) and presence of distant metastasis were independent poor prognostic factors with a hazards ratio of 2.20 (*p = 0*.*008*) and 3.1 (*p = 0*.*01*), respectively (Table 5). Although CD4+ and FOXP3+ TILs were a significantly favorable prognostic factor by the univariate analysis, the multivariate analysis did not show statistical significance for TILs. Furthermore, even if CD8+, CD4+, and FOXP3+ TILs were scored same as Immunoscore (TILs score; 0–3), TILs score was not an independent prognostic factor for OS and PFS (a hazards ration of 0.59, *p = 0*,*053* and 0.71, *p = 0*.*22*, respectively: S1 Table).

**Table 4:** Univariate and multivariate cox regression analysis for overall survival in 130 patients with BTC.

		Univariate			Multivariate		
		HR	95% CI	P value	HR	95% CI	P value
T category	pT≧3	1.76	1.06–2.91	*0.028	1.36	0.76–2.42	0.3
Lymph node metastasis		2.71	1.63–4.49	*<0.001	2.0	1.08–3.66	*0.02
Distant metastasis		5.31	2.37–10.7	*<0.001	3.34	1.42–7.32	*0.007
Serum CEA level	≧5 ng/ml	1.62	0.83–2.93	0.14			
Serum CA19-9 level	≧37 U/ml	2.0	1.17–3.48	*0.01	1.72	1.00–3.01	*0.04
CD8 TILs	High	0.72	0.44–1.19	0.2			
CD4 TILs	High	0.56	0.34–0.95	*0.03	0.66	0.37–1.18	0.16
FOXP3 TILs	High	0.63	0.35–1.08	0.1			
CD68 TAMs	High	1.66	0.96–2.76	0.05			

*p < 0.05

BTC: biliary tract cancers, CEA; carcinoembryonic antigen, CA19-9; carbohydrate antigen 19–9, TILs; tumor infiltrating lymphocytes, TAMs; tumor associated macrophages, HR; Hazards ration, CI; confidence interval.

**Table 5:** Univariate and multivariate cox regression analysis for recurrence free survival in 130 patients with BTC.

		Univariate			Multivariate		
		HR	95% CI		HR	95% CI	P value
T category	pT≧3	2.36	1.44–3.89	*<0.001	2.20	1.22–3.99	*0.008
Lymph node metastasis		2.99	1.82–4.91	*<0.001	1.85	0.99–3.4	0.05
Distant metastasis		5.80	2.61–11.6	*<0.001	3.1	1.25–7.18	*0.01
Serum CEA level	≧5 ng/ml	2.16	1.12–3.91	*0.02	1.72	0.85–3.27	0.13
Serum CA19-9 level	≧37 U/ml	1.99	1.19–3.37	*0.008	1.5	0.88–2.59	0.13
CD8 TILs	High	0.68	0.42–1.11	0.12			
CD4 TILs	High	0.43	0.27–0.72	*0.001	0.57	0.33–1.03	0.06
FOXP3 TILs	High	0.57	0.33–0.97	*0.03	0.73	0.41–1.27	0.27
CD68 TAMs	High	1.47	0.88–2.42	0.13			

*p < 0.05

BTC: biliary tract cancers, CEA; carcinoembryonic antigen, CA19-9; carbohydrate antigen 19–9, TILs; tumor infiltrating lymphocytes, TAMs; tumor associated macrophages, HR; Hazards ration, CI; confidence interval.

### Association between Immunoscore and survival outcomes

**Fig 4:**
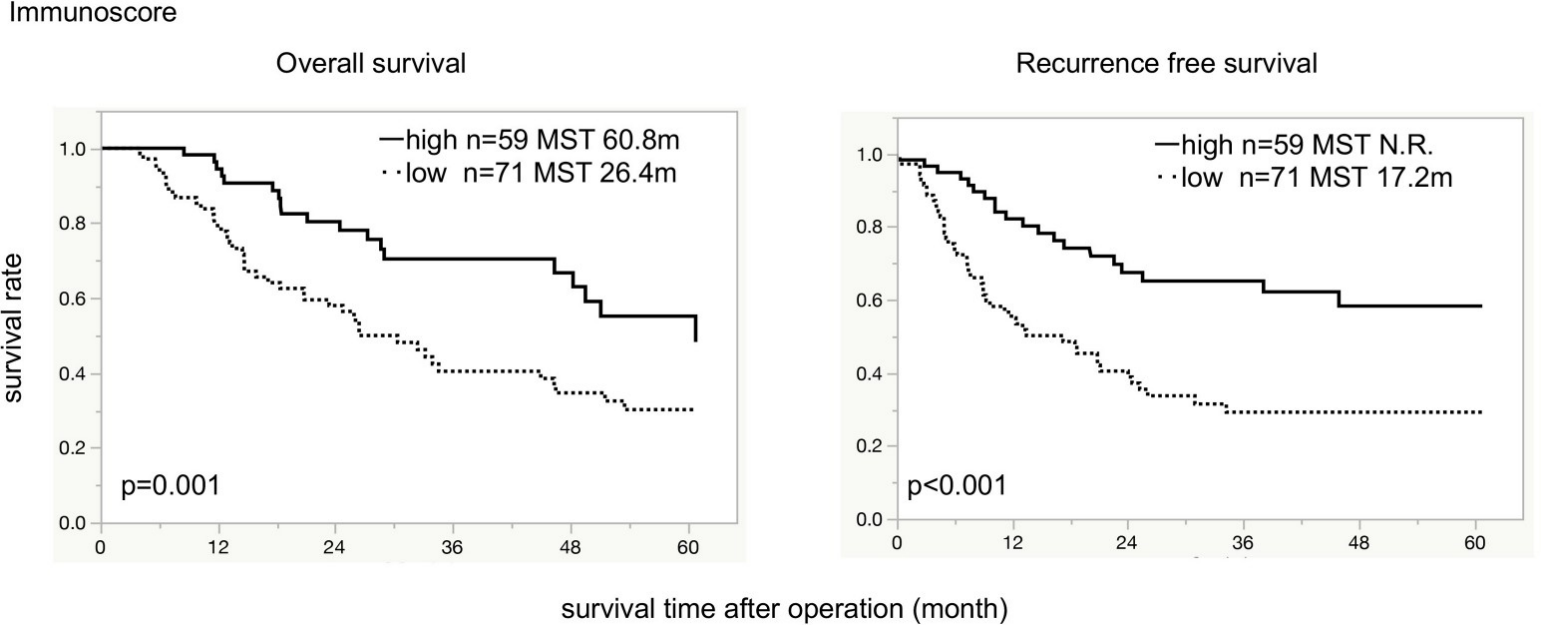
Overall survival and recurrence-free survival for tumor-infiltrating immune cells score. Immunoscore was assigned a number from 0 to 4, according to CD8+ high, CD4+ high, FOXP3+ high, and CD68+ low-infiltration. Kaplan-Meier survival curve indicates that the high Immunoscore group showed significantly superior OS and RFS than the low Immunoscore group. OS: overall survival, RFS: recurrence-free survival, MST: median survival time, N.R.: not reached.

To understand the influence of immune cells infiltration into TME on tumor progression, we evaluated the Immunoscore based on the status of infiltrating immune cells. The number of cases was 18 for score 4, 41 for score 3, 40 for score 2, 29 for score 1, and 2 for score 0. A total of 59 cases had high Immunoscore (3–4) and 71 cases had low Immunoscore (0–2). Patients with high Immunoscore showed significantly superior OS and RFS than those with low Immunoscore (median OS 60.8 vs. 26.4 months, respectively, *p = 0*.*001*; median RFS not reached vs. 17.2 months, respectively, *p < 0*.*001*; Fig 4).

### Multivariate analysis of Immunoscore

Multivariate analysis of the patients was performed with clinicopathological predictors and Immunoscore (Table 2). For OS, low Immunoscore, positive lymph node metastasis, presence of distant metastasis, and high serum CA19-9 level were independent poor prognostic factors (hazards ratio 2.05, 3.48, 1.7, and 2.05, respectively; *p = 0*.*01*, *p = 0*.*005*, *p = 0*.*05*, and *p = 0*.*01*, respectively). For RFS, low Immunoscore, T classification of pT3-4, and presence of distant metastasis were independent poor prognostic factors with a hazards ratio of 2.41 (*p = 0*.*001*), 2.16 (*p = 0*.*005*), and 3.58 (*p = 0*.*005*), respectively (Table 6).

**Table 6:** Multivariate cox regression analysis for overall and recurrence free survival in 130 patients with BTC.

		Multivariate for OS			Multivariate for RFS		
		HR	95% CI	P value	HR	95% CI	P value
T category	pT ≧ 3	1.35	0.76–2.39	0.28	2.16	1.26–3.76	*0.005
Lymph node metastasis		2.07	1.11–3.78	*0.02	1.78	0.97–3.21	0.06
Distant metastasis		3.48	1.47–7.68	*0.005	3.58	1.49–8.01	*0.005
Serum CA19-9 level	≧37 U/ml	1.7	0.99–2.97	*0.05	1.55	0.92–2.63	0.09
Immunoscore	≧3	2.05	1.18–3.67	*0.01	2.41	1.41–4.23	*0.001

*p < 0.05

BTC: biliary tract cancers, CA19-9; carbohydrate antigen 19–9, OS; overall survival, RFS; recurrence free survival, HR; Hazards ration, CI; confidence interval.

## Discussion

This study indicated that although TILs, including CD8+ T cells, CD4+ T cells, and FOXP3+ T cells, did not correlate with clinicopathological factors and each T cell alone was not an independent prognostic factor, TILs tended to improve the prognosis of patients with BTC. On the other hand, high-infiltration with macrophages did not show a poor prognosis, and it is also not a significant independent prognostic factor for OS and RFS. When every infiltrating T cell and macrophage in TME were comprehensively scored, a high Immunoscore group had significantly longer OS and RFS and was an independent prognostic factor in the multivariate analysis. Our findings indicated that in BTC, evaluating TILs and TAMs in TME comprehensively was better than evaluating one TIIC, such as T cell or macrophage, each by each.

TILs are composed of T cells, B cells, and natural killer (NK) cells [21]. Among them, T cells play the most central role in TME. In the current study, we evaluated CD8+ T cell, CD4+ T cell, and regulatory T cells. CD8+ T cells have been reported to have several subsets such as naïve CD8 T cell, memory CD8 T cell, and effector CD8 T cell [22]. The effector CD8 T cells are well-known and play a cytotoxic role to attack tumors directly [23]. Many researchers revealed a correlation between infiltration of CD8+ TILs and survival outcomes [24]. Moreover, CD8+ TILs can also be useful for predicting the effects of immunotherapy [25, 26]. We also reported an association between CD8+ infiltration and patient outcome in BTC [20]. Unlike CD8+ TILs, CD4+ TILs might contribute to anti-tumor immunity via cytokines [27, 28]. However, the function of CD4+ TILs in TME is still unclear due to the existence of many subsets [29]. In the current study, CD4+ TILs helped anti-tumor immunity. Regulatory T cells (Tregs) were initially characterized as CD4+/CD25+ T cells, and Tregs cell markers are known as FOXP3 [30]. FOXP3+ T cell might suppress the activity of cytotoxic T cells via cytokines; therefore, high-infiltration with FOXP3+ TILs is correlated with poor prognosis in several cancers [31, 32]. However, some researchers indicated that FOXP3 is one of the unfavorable prognostic factors in colorectal cancer [33]. The reason of these discrepancies is that the role of immune cells in the microenvironment differs depending on the origin of the tumor [34]. FOXP3+ TILs suppress tumor-promoting inflammatory responses under the presence of the enteric bacteria [33]. That’s why high-infiltration with FOXP3+ is not always associated the good prognostic factor, and similar reacts may occur in BTC. There are many previous reports about TILs, however, in TME, each immune cell interacts with the other. We considered that it is necessary to evaluate immune cells comprehensively as TME.

Basically, TAMs are involved in tumor progression. However, in a meta-analysis, high-infiltrating TAMs were associated with a poor prognosis in gastric, breast, and ovarian cancer whereas these were associated with a good prognosis in colorectal cancer [9]. The reason for this discrepancy is that macrophages have two main phenotypes, M1 and M2. M1 macrophages induced by cytokines, such as transforming growth factor (TGF)-β, interleukin (IL)-6, and IL-10, have anti-tumor activity. On the other hand, M2 macrophages induced by IL-4 and IL-13 play a key role in tumor progression and metastasis [35]. CD68 is known as a pan-macrophage marker and is correlated with a poor prognosis in breast cancer and lymphoma [36–38]. In this study, CD68 was used as a marker for TAMs instead of CD80 for M1, CD163 or CD206 for M2 specific macrophage marker [39]. Therefore, high-infiltration with CD68+ TAMs was not correlated with tumor progression. To evaluate tumor progression with each specific marker, it might be useful to investigate the effect of macrophages on patient outcomes. Furthermore, although the infiltration of CD68+ TAMs did not show correlation with the infiltration of TILs in our current study, it has been reported that TAMs suppress immune reaction to tumors in TME [40]. Further evaluation of immunosuppression markers with iNOS or IDO may help understand the function of immune cells in TME.

We investigated whether it is possible to comprehensively assess T-cell infiltration using CD3 marker as a pan-T cell marker. Cases with the number of infiltrating CD3+ TILs that varied from 0 to 480/high power field (cut-off 52) (S4A Fig) and 52.3% (68 out of 130 cases) were assigned to the high-infiltration CD3+ TILs group (S4B and S4C Fig). The high-infiltration CD3+ TILs group showed significantly superior RFS than the low-infiltration CD3+ TILs group. For OS, there is no significant difference between the high- and low- infiltration CD3+ TILs groups (median OS 46.4 vs. 45 months, respectively, *p = 0*.*08*; median RFS 34.2 vs. 17.3 months, respectively, *p = 0*.*03*) (S4D Fig). Comprehensive evaluation with CD3+ TILs and CD68+ TAMs showed the low-infiltration CD3+ TILs and high-infiltration CD68+ TAM group was an independent poor prognostic factor in only OS (others vs. CD3+ low and CD68+ high; median OS 48.3 vs 20.8 months, *p = 0*.*01*; hazards ratio 2.65, *p = 0*.*01*), but not in RFS (median RFS 26.1 vs. 11.7 months, *p = 0*.*09*; hazards ratio 1.54, *p = 0*.*24*) (S4E Fig, S2 Table). Immunoscore, which is a score to evaluate each immune cell infiltration, was more associated with OS and RFS. This indicates that evaluating various subsets of immune cells in TME would be a better prediction factor of OS and RFS. However, evaluating all subsets by immunohistochemical staining with each marker are complicated and may not be feasible. Therefore, to apply the results obtained in our study to clinical in the future, more convenient and simple evaluation method will be necessary, such as evaluating immune cell infiltration with only H & E staining [41].

Not only malignancy of the tumor itself but also tumor immunity in TME, including TILs and TAMs, B cells, NK cells, neutrophils, and dendric cells, is involved in tumor progression and patient outcomes [42]. Recently, various types of therapeutic strategies targeting immune cells have been developed, including adoptive cell therapy, TIL therapy, T cell receptor gene therapy, chimeric antigen receptor (CAR) T cell therapy, NK cell therapy, and CAR NK cell therapy [43–45]. Nowadays, several clinical trials on adoptive T cell therapy for BTC are ongoing [46, 47]. We believe that our findings contribute to the development and selection of a treatment strategy for BTC.

There are some limitations to this study. First, this study was a retrospective single-center cohort. Retrospective nature limits our understanding of the associations while single-center nature limits the generalizability of the findings. Second, there is not validation cohorts in this study to check the cutoff value are appropriate or not. Third, it included only five subtypes of BTCs, i.e., intrahepatic, perihilar, and distal bile duct cancer, gallbladder cancer, and ampullary cancer. This limits the application of findings to other cancer types. Fourth, we did not analyze other immune cells by immunohistochemical staining, such as B cells, neutrophils, or dendric cells. These immune cells may also affect tumor progression in TME. Lastly, we did not clarify the polarization of the macrophages. CD68 was initially proposed to exclude macrophages, however, it has recently been reported to be expressed in dendritic cells, tumor cells, endothelial cells, and fibroblasts.

In conclusion, we found that the high Immunoscore group had significantly longer OS and RFS and was an independent prognostic factor. Our findings indicated that in biliary tract cancer, the evaluation of infiltrating immune cells in TME was useful to predict patient prognosis.

## Supporting information

S1 Fig H & E staining of tissue micro array. (TIF)

S2 Fig The number of infiltrating immune cells. HPF; high power field.
(TIF)

S3 Fig Receiver operating characteristic (ROC) curve analyses for 5-year RFS. TILs; tumor-infiltrating lymphocytes, TAMs; tumor associated macrophages. AUC; area under the curve.
(TIF)

S4 Fig The impact of CD3+ TILs infiltration. a: The number of infiltrating CD3+ TILs. b, c: IHC staining with CD3 antibody. b: low infiltration, c: high infiltration. d: overall survival and recurrence-free survival for CD3+ TILs. e: overall survival and recurrence-free survival for CD3+ TILs and CD68+ TAMs. HPF; high power field, IHC; immunohistochemical staining, TILs; tumor-infiltrating lymphocytes, TAMs; tumor associated macrophages.
(TIF)

S1 Table Multivariate cox regression analysis for overall and recurrence free survival in 130 patients with BTC. (DOCX)

S2 Table Multivariate cox regression analysis for overall and recurrence free survival in 130 patients with BTC. (DOCX)

## References

[1] Hanahan D, Coussens LM. Accessories to the Crime: Functions of cells Recruited to the Tumor Microenvironment. Cancer Cell 2012;21: 309–322. doi: 10.1016/j.ccr.2012.02.022 22439926

[2] Fridman WH, Pages F, Sautes-Fridman C, Galon J. The immune contexture in human tumours: impact on clinical outcome. Nat Rev Cancer 2021;12: 298–306. 10.1038/nrc3245 22419253

[3] Iseki Y, Shibutani M, Maeda K, Nagahara H, Fukuoka T, Matsutani S, et al. A new method for evaluating tumor-infiltrating lymphocytes (TILs) in colorectal cancer using hematoxylin and eosin (H-E)-stained tumor sections. PLoS One 2018;13: e0192744. doi: 10.1371/journal.pone.0192744 29698402 PMC5919485

[4] Goto W, Kashiwagi S, Asano Y, Takada K, Takahashi K, Hatano T, et al. Predictivevalue of improvement in the immune tumour microenvironment in patients with breast cancer treated with neoadjuvant chemotherapy. ESMO Open 2018;3, e000305. doi: 10.1136/esmoopen-2017-000305 30233820 PMC6135412

[5] Shibutani M, Maeda K, Nagahara H, Fukuoka T, Nakao S, Matsutani S, et al. The peripheral monocyte count is associated with the density of tumor-associated macrophages in the tumor microenvironment of colorectal cancer: a retrospective study. BMC Cancer 2017;17: 404. doi: 10.1186/s12885-017-3395-1 28583114 PMC5460583

[6] Shibutani M, Maeda K, Nagahara H, Fukuoka T, Iseki Y, Matsutani S, et al. Tumor-infiltrating Lymphocytes Predict the Chemotherapeutic Outcome in Patients with Stage IV Colorectal Cancer. In Vivo 2018;32: 151–158. 29275313 10.21873/invivo.11218 PMC5892643

[7] Zhang Y, Zhang Z. The history and advances in cancer immunotherapy: understanding the characteristic of tumor-infiltrating immune cells and their therapeutic implications. Cell Mol Immunol 2020;17: 807–821. 32612154 10.1038/s41423-020-0488-6 PMC7395159

[8] Noy R, Pollard JW. Tumor-associated macrophages: from mechanisms to therapy. Immunity 2014;41: 49–61. doi: 10.1016/j.immuni.2014.06.010 25035953 PMC4137410

[9] Zhang Q, Liu L, Gong C, Shi H, Zeng Y, Wang X, et al. Prognostic Significance of Tumor-Associated Macrophages in Solid Tumor: A Meta-Analysis of the Literature. PLoS One 2012;7: e50946. doi: 10.1371/journal.pone.0050946 23284651 PMC3532403

[10] Gou M, Zhang Y, Si H, Dai G. Efficacy and safety of nivolumab for metastatic biliary tract cancer. Onco Targets Ther. 2019;12: 861–867. doi: 10.2147/OTT.S195537 30774373 PMC6355165

[11] Kitano Y, Okabe H, Yamashita Y, Nakagawa S, Saito Y, Umezaki N, et al. Tumour-infiltrating inflammatory and immune cells in patients with extrahepatic cholangiocarcinoma. Br J Cancer 2018;118: 171–180. doi: 10.1038/bjc.2017.401 29123259 PMC5785749

[12] Goeppert B, Frauenschuh L, Zucknick, Stenzinger A, Andrulis M, Klauschen, et al. Prognostic impact of tumour-infiltrating immune cells on biliary tract cancer. Br J Cancer 2013;109: 2665–2674. doi: 10.1038/bjc.2013.610 24136146 PMC3833207

[13] Miura T, Yoshizawa T, Hirai H, Seino H, Morohashi S, Wu Y, et al. Prognostic Impact of CD163^+^ Macrophages in Tumor Stroma and CD8^+^ T-Cells in Cancer Cell Nests in Invasive Extrahepatic Bile Duct Cancer. Anticancer Res. 2017;37: 183–190. 28011489 10.21873/anticanres.11304

[14] Hasita H, Komohara Y, Okabe H, Masuda T, Ohnishi K, Lei XF, et al. Significance of alternatively activated macrophages in patients with intrahepatic cholangiocarcinoma. Cancer Sci. 2010;101: 1913–1919. doi: 10.1111/j.1349-7006.2010.01614.x 20545696 PMC11158749

[15] Atanasov G, Hau HM, Dietel C, Benzing C, Krenzien F, Brandl A, et al. Prognostic significance of macrophage invasion in hilar cholangiocarcinoma. BMC Cancer 2015;15: 790. doi: 10.1186/s12885-015-1795-7 26497197 PMC4620011

[16] Nakakubo Y, Miyamoto M, Cho Y, Hida Y, Oshikiri T, Suzuoki M, et al. Clinical significance of immune cell infiltration within gallbladder cancer. Br J Cancer 2003;89: 1736–1742. doi: 10.1038/sj.bjc.6601331 14583778 PMC2394404

[17] Miyazaki M, Ohtsuka M, Miyakawa S, Nagino M, Yamamoto M, Kokubo N, et al. Classification of biliary tract cancers established by the Japanese Society of Hepato-Biliary-Pancreatic Surgery: 3(rd) English edition. J Hepatobiliary Pancreat Sci. 2015;22: 181–196. doi: 10.1002/jhbp.211 25691463

[18] Amin MB, Greene FL, Edge SB, Compton CC, Gershenwald JF, Brookland RK, et al. The Eighth Edition AJCC Cancer Staging Manual: Continuing to build a bridge from a population-based to a mor “personalized” approach to cancer staging. CA Cancer J Clin. 2017;67: 93–99. 28094848 10.3322/caac.21388

[19] Matsutani S, Shibutani M, Maeda K, Nagahara H, Fukuoka T, Nakao S, et al. Significance of tumor-infiltrating lymphocytes before and after neoadjuvant therapy for rectal cancer. Cancer Sci. 2018;109: 966–979. doi: 10.1111/cas.13542 29464828 PMC5891199

[20] Tanaka R, Kimura K, Eguchi S, Tauchi J, Shibutani M, Shinkawa H, et al. Preoperative Neutrophil-to-lymphocyte Ratio Predicts Tumor-infiltrating CD8^+^ T Cells in Biliary Tract Cancer. Anticancer Res. 2020;40: 2881–2887. 32366438 10.21873/anticanres.14264

[21] Mao Y, Qu Q, Chen X, Huang O, Wu J, Shen K. The Prognostic Value of Tumor-Infiltrating Lymphocytes in Breast Cancer: A Systematic Review and Meta-Analysis. PLoS One 2016;11: e0152500. doi: 10.1371/journal.pone.0152500 27073890 PMC4830515

[22] Dolina JS, Braeckel-Budimir NV, Thomas GD, Salek-Ardakani S. CD8+ T Cell Exhaustion in Cancer. Front Immunol. 2021;12: 715234. doi: 10.3389/fimmu.2021.715234 34354714 PMC8330547

[23] Maimela NR, Liu S, Zhang Y. Fates of CD8+ T cells in Tumor Microenvironment. Comput Struct Biotechnol J. 2018;17: 1–13. doi: 10.1016/j.csbj.2018.11.004 30581539 PMC6297055

[24] Jochems C, Schlom J. Tumor-infiltrating immune cells and prognosis: the potential link between conventional cancer therapy and immunity. Exp Biol Med. (Maywood) 2011;236: 567–579. doi: 10.1258/ebm.2011.011007 21486861 PMC3229261

[25] Loupakis F, Depetris I, Biason P, Intini R, Prete AA, Leone F, et al. Prediction of Benefit from Checkpoint Inhibitors in Mismatch Repair Deficient Metastatic Colorectal Cancer: Role of Tumor Infiltrating Lymphocytes. Oncologist 2020;25: 481–487. doi: 10.1634/theoncologist.2019-0611 31967692 PMC7288636

[26] Stanton SE, Disis ML. Clinical significance of tumor-infiltrating lymphocytes in breast cancer. J Immunother Cancer 2016;4: 59. doi: 10.1186/s40425-016-0165-6 27777769 PMC5067916

[27] Friedman KM, Prieto PA, Devillier LE, Gross CA, Yang JC, Wunderlich JR, et al. Tumor-specific CD4+ melanoma tumor-infiltrating lymphocytes. J Immunother. 2012;35: 400–408. doi: 10.1097/CJI.0b013e31825898c5 22576345 PMC7412749

[28] Borst J, Ahrends T, Babata N, Melief CJM, Kastenmuller W. CD4+ T cell help in cancer immunology and immunotherapy. Nat Rev Immunol. 2018;18: 635–647. doi: 10.1038/s41577-018-0044-0 30057419

[29] Kim HJ, Cantor H. CD4 T-cell subsets and tumor immunity: the helpful and the not-so-helpful. Cancer Immunol Res. 2014;2: 91–98. doi: 10.1158/2326-6066.CIR-13-0216 24778273

[30] Salama P, Phillips M, Grieu F, Morris M, Zeps N, Joseph D, et al. Tumor-infiltrating FOXP3+ T regulatory cells show strong prognostic significance in colorectal cancer. J Clin Oncol. 2009;27: 186–192. doi: 10.1200/JCO.2008.18.7229 19064967

[31] Kobayashi N, Hiraoka N, Yamagami W, Ojima H, Kanai Y, Kosuge T, et al. FOXP3+ regulatory T cells affect the development and progression of hepatocarcinogenesis. Clin Cancer Res. 2007;13: 902–911. doi: 10.1158/1078-0432.CCR-06-2363 17289884

[32] Hiraoka N, Onozato K, Kosuge T, Hirohashi S. Prevalence of FOXP3+ regulatory T cells increases during the progression of pancreatic ductal adenocarcinoma and its premalignant lesions. Clin Cancer Res. 2006;12: 5423–5434. doi: 10.1158/1078-0432.CCR-06-0369 17000676

[33] Ladoire S, Martin F, Ghiringhelli F. Prognostic role of FOXP3+ regulatory T cells infiltrating human carcinomas: the paradox of colorectal cancer. Cancer Immunol Immunother. 2011;60: 909–918. doi: 10.1007/s00262-011-1046-y 21644034 PMC11028605

[34] deLeeuw RJ, Kost SE, Kakal JA, Nelson BH. The prognostic value of FOXP3+ tumor-infiltrating lymphocytes in cancer: a critical review of the literature. Clin Cancer Res. 2012;18: 3022–3029. doi: 10.1158/1078-0432.CCR-11-3216 22510350

[35] Allavena P, Sica A, Solinas G, Porta C, Mantovani A. The inflammatory micro-environment in tumor progression: the role of tumor-associated macrophages. Crit Rev Oncol Hematol. 2008;66: 1–9. doi: 10.1016/j.critrevonc.2007.07.004 17913510

[36] Jeong H, Hwang I, Kang SH, Shin HC, Kwon SY. Tumor-Associated Macrophages as Potential Prognostic Biomarkers of Invasive Breast Cancer. J Breast Cancer 2019;22: 38–51. doi: 10.4048/jbc.2019.22.e5 30941232 PMC6438840

[37] Cai Q, Liao H, Lin S, Xia Y, Wang X, Gao Y, et al. High expression of tumor-infiltrating macrophages correlates with poor prognosis in patients with diffuse large B-cell lumphoma. Med Oncol. 2012;29: 2317–2322. 22198695 10.1007/s12032-011-0123-6

[38] Medrek C, Ponten F, Jirstrom K, Leandersson K. The presence of tumor associated macrophages in tumor stroma as a prognostic marker for breast cancer patients. BMC Cancer 2012;12: 306. doi: 10.1186/1471-2407-12-306 22824040 PMC3414782

[39] Bertani FR, Mozetic P, Fioramonti M, Iuliani M, Ribelli G, Pantano F, et al. Classification of M1/M2-polarized human macrophages by label-free hyperspectral reflectance confocal microscopy and multivariate analysis. Sci Rep. 2017;7: 8965. doi: 10.1038/s41598-017-08121-8 28827726 PMC5566322

[40] DeNardo DG, Ruffell B. Macrophages as regulators of tumour immunity and immunotherapy. Nat Rev Immunol. 2019;19: 369–382. doi: 10.1038/s41577-019-0127-6 30718830 PMC7339861

[41] Matsutani S, Shibutani M, Maeda K, Nagahara H, Fukuoka T, Iseki Y, et al. Tumor-infiltrating Immune Cells in H&E-stained Sections of Colorectal Cancer Tissue as a Reasonable Immunological Biomaker. Anticancer Res. 2018;38: 6721–6727. 30504382 10.21873/anticanres.13041

[42] Upadhyay S, Sharma N, Gupta KB, Dhiman M. Role of immune system in tumor progression and carcinogenesis. J Cell Biochem. 2018;119: 5028–5042. doi: 10.1002/jcb.26663 29327370

[43] Rohaan MW, Wilgenhof S, Haanen JBAG. Adoptive cellular therapies: the current landscape. Virchows Arch. 2019;474: 449–461. doi: 10.1007/s00428-018-2484-0 30470934 PMC6447513

[44] Du N, Guo F, Wang Y, Cui J. NK Cell Therapy: A Rising Star in Cancer Treatment. Cancers (Basel) 2021;13: 4129. doi: 10.3390/cancers13164129 34439285 PMC8394762

[45] Vacca P, Pietra G, Tumino N, Munari E, Mingari MC, Moretta L. Exploiting Human NK Cells in Tumor Therapy. Front Immunol. 2020;10: 3013. doi: 10.3389/fimmu.2019.03013 32010130 PMC6978749

[46] Loeuillard E, Conboy CB, Gores GJ, Rizvi S. Immunobiology of cholangiocarcinoma. JHEP Rep. 2019;1: 297–311. doi: 10.1016/j.jhepr.2019.06.003 32039381 PMC7001542

[47] Guo Y, Feng K, Liu Y, Wu Z, Dai H, Yang Q, et al. Phase I Study of Chimeric Antigen Receptor-Modified T Cells in Patients with EGFR-Positive Advanced Biliary Tract Cancers. Clin Cancer Res. 2018;24: 1277–1286. doi: 10.1158/1078-0432.CCR-17-0432 29138340

