# Supplementary figures and images for: Tumor-infiltrating lymphocytes and macrophages as a significant prognostic factor in biliary tract cancer
Source: PLoS One. 2023 Jan 24;18(1):e0280348. doi: 10.1371/journal.pone.0280348 (PMC9873170; doi:10.1371/journal.pone.0280348)

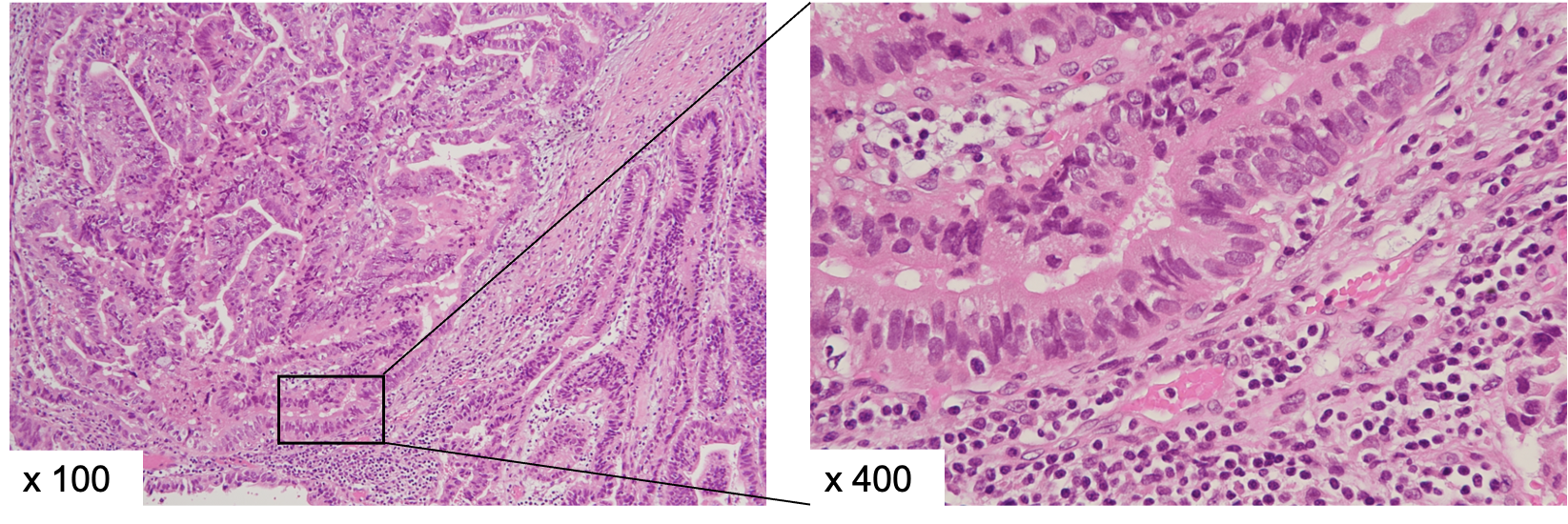

Supplement: S1 Fig — (TIF) [file pone.0280348.s001.tif]

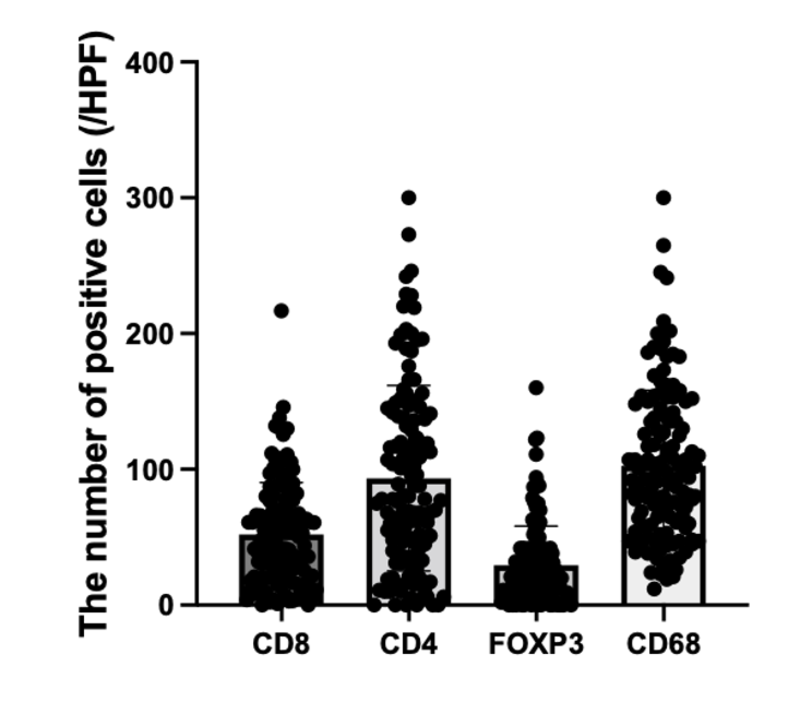

Supplement: S2 Fig — HPF; high power field. (TIF) [file pone.0280348.s002.tif]

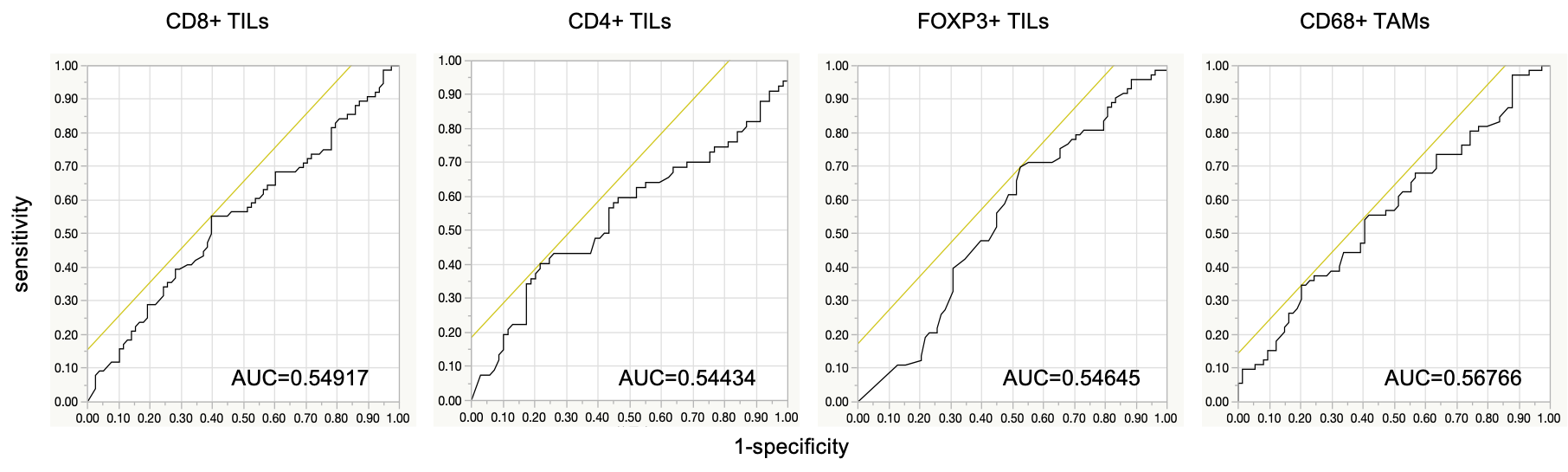

Supplement: S3 Fig — TILs; tumor-infiltrating lymphocytes, TAMs; tumor associated macrophages. AUC; area under the curve. (TIF) [file pone.0280348.s003.tif]

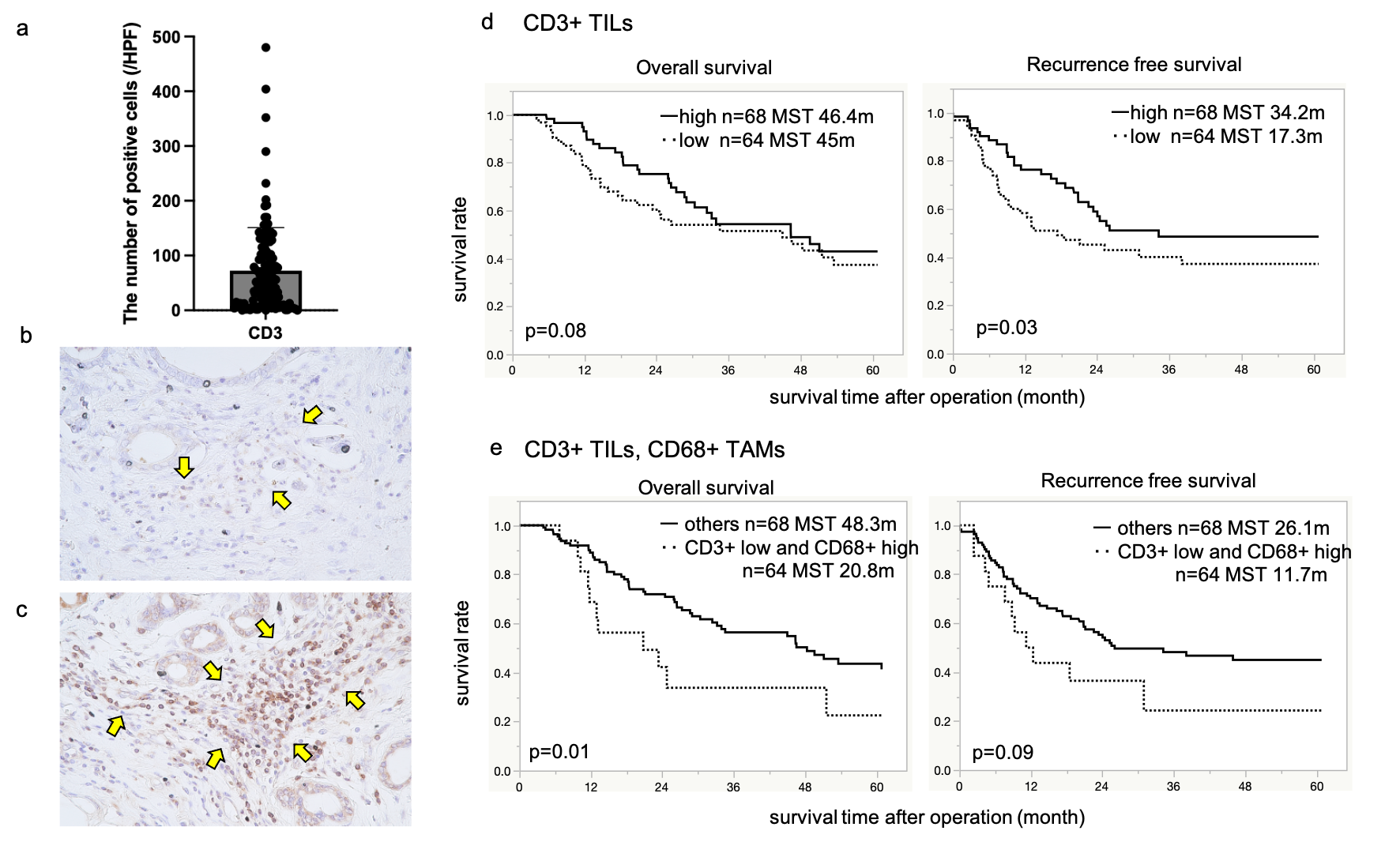

Supplement: S4 Fig — a: The number of infiltrating CD3+ TILs. b, c: IHC staining with CD3 antibody. b: low infiltration, c: high infiltration. d: overall survival and recurrence-free survival for CD3+ TILs. e: overall survival and recurrence-free survival for CD3+ TILs and CD68+ TAMs. HPF; high power field, IHC; immunohistochemical staining, TILs; tumor-infiltrating lymphocytes, TAMs; tumor associated macrophages. (TIF) [file pone.0280348.s004.tif]
