# Supplementary material for: Tumor-infiltrating lymphocytes and macrophages as a significant prognostic factor in biliary tract cancer
Source: PLoS One. 2023 Jan 24;18(1):e0280348. doi: 10.1371/journal.pone.0280348 (PMC9873170; doi:10.1371/journal.pone.0280348)
Supplement: S1 Table — (DOCX) [file pone.0280348.s005.docx]

**S1 Table.** Multivariate cox regression analysis for overall and recurrence free survival in 130 patients with BTC.

|  |  | Multivariate for OS | | |  | Multivariate for RFS | | |
| --- | --- | --- | --- | --- | --- | --- | --- | --- |
|  |  | HR | 95% CI | P value |  | HR | 95% CI | P value |
| T category | pT ≧ 3 | 1.48 | 0.83–2.68 | 0.18 |  | 2.20 | 1.25–3.98 | *0.006 |
| Lymph node metastasis | | 1.98 | 1.07–3.62 | *0.03 |  | 1.55 | 0.84–2.82 | 0.15 |
| Distant metastasis |  | 3.41 | 1.44–7.51 | *0.006 |  | 3.22 | 1.34–7.19 | *0.01 |
| Serum CA19-9 level | ≧37 U/ml | 1.66 | 0.96–2.93 | 0.07 |  | 1.53 | 0.91–2.62 | 0.11 |
| TILs score (CD8+, CD4+, and FOXP3+) | ≧2 | 0.71 | 0.41–1.23 | 0.22 |  | 0.59 | 0.36–1.00 | 0.053 |

^*^p < 0.05

BTC: biliary tract cancer, CA19-9; carbohydrate antigen 19-9, TILs; tumor-infiltrating lymphocytes, OS; overall survival, RFS; recurrence free survival, HR; Hazards ration, CI; confidence interval.
