# Supplementary material for: Tumor-infiltrating lymphocytes and macrophages as a significant prognostic factor in biliary tract cancer
Source: PLoS One. 2023 Jan 24;18(1):e0280348. doi: 10.1371/journal.pone.0280348 (PMC9873170; doi:10.1371/journal.pone.0280348)
Supplement: S2 Table — (DOCX) [file pone.0280348.s006.docx]

**S2 Table.** Multivariate cox regression analysis for overall and recurrence free survival in 130 patients with BTC.

|  |  | Multivariate for OS | | |  | Multivariate for RFS | | |
| --- | --- | --- | --- | --- | --- | --- | --- | --- |
|  |  | HR | 95% CI | P value |  | HR | 95% CI | P value |
| T category | pT ≧ 3 | 1.34 | 0.75–2.43 | 0.31 |  | 2.15 | 1.19–3.97 | *0.01 |
| Lymph node metastasis | | 2.08 | 1.11–3.84 | *0.02 |  | 1.79 | 0.95–3.34 | 0.06 |
| Distant metastasis |  | 3.74 | 1.51–8.47 | *0.006 |  | 3.79 | 1.52–8.67 | *0.005 |
| Serum CA19-9 level | ≧37 U/ml | 1.72 | 0.99–3.04 | 0.05 |  | 1.66 | 0.97–2.89 | 0.06 |
| Low CD3+ TILs and high CD68+ TAMs |  | 2.65 | 1.18–5.35 | *0.02 |  | 1.54 | 0.69–3.05 | 0.26 |

^*^p < 0.05

BTC: biliary tract cancer, CA19-9; carbohydrate antigen 19-9, TILs; tumor-infiltrating lymphocytes, TAMs; tumor associated macrophages, OS; overall survival, RFS; recurrence free survival, HR; Hazards ration, CI; confidence interval.
