# Supplementary material for: Tumor-infiltrating lymphocytes and macrophages as a significant prognostic factor in biliary tract cancer
Source: PLoS One. 2023 Jan 24;18(1):e0280348. doi: 10.1371/journal.pone.0280348 (PMC9873170; doi:10.1371/journal.pone.0280348)
Supplement: Attachment — Submitted filename: Response to Reviews.docx [file pone.0280348.s007.docx]

Point by point response to Reviewer 1 Comments

We are grateful for your comments which have helped to improve our manuscript. As indicated in the responses that follow, we have taken your comments into account in our manuscript.

*1. In the Figure 1, the authors shown representative immunohistochemistry images of high and low infiltration of CD8-high, CD4-high, and FOXP3-high TILs and CD68-low CD8+, CD4+, FOXP3+, and CD68+ cells, in human patients. First, the authors need to mention the kind of microscope by which these pictures are taken. Also, they please a graph shown a numerical evaluation of the staining it should be appreciable by the readers. Furthermore, need to add some arrows to show the infiltration of the above cells in the tissue. In the Figure1, the authors consider two groups of patients with low and high infiltration, it should be interesting if in the Figure 1 they add also images of the healthy control group.*

Response: We added the information of microscope in page 9, line 92-93. We added the figure of the number of infiltrating immune cells as Supplementary Figure 2.

We added arrows to the stained immune cells in Figure 1.

In this current study, we evaluated the infiltration of immune cells into the tumor microenvironment in patients with biliary tract cancer. Also, we investigated how well the infiltration of immune cells can predict clinical prognosis. Because healthy patients do not have tumor, it is impossible to evaluate immune cell infiltrations into their tumor microenvironment. In addition, it is difficult to obtain biliary tract specimens from healthy non-cancer-bearing patients.

*2. In the Table1, the authors listed some of the clinicopathological characteristics of the 130 patients with BTC. Please, the authors must complete the list, give some more info such as age, eventually pharmacological treatment of the patients, stage of the illness ecc.*

Response: We already listed age and stage in Table 1. We added the information of pharmacological treatment into Table 1.

*3. In the << tissue microarray construction>> paragraph, the authors ensured that representative tumor cell-rich areas are H&E stained with a light microscope and were sent to create TMA blocks. The authors must include some H&E images of the tumor sections in their results. So the readers can appreciate the percentage of TILs on H&E stained samples.*

Response: We added the images of H&E staining as Supplementary Figure 1.

*4.* *Is well known that immunohistochemical markers used to identify M1 and M2 TAMs are the keystones of TAM evaluation. In this manuscript the authors study the tumor-associated macrophages (TAMs) in BTC. It should be interesting if the authors extent their experiments (immunohistochemistry or PCR or blots) in other markers that characterized the TAMs, such as, CD11c, CD86, iNOS, pSTAT1 (M1 markers), CD163, CD204, CD206 (M2 markers) or at least some of them.*

Response: I totally agree with your point. Performing double staining using various cell surface markers will allow us to understand immune cell infiltration in TME in more detail. However, our future goal is to examine how to apply this result to clinical practice, and we would like to replace it with a simple method. We evaluated CD3 positive cells as a pan-T cell marker. We found that CD3 is useful to understand the infiltration of immune cells in TME, although the accuracy decreased compared to each immune cell evaluation. In the future, we will investigate whether predicting prognosis is possible by evaluating immune cell infiltration with H & E staining. We added the paragraph in page 31-32, line 305-325. Also, we added Supplementary Figure 4 and Supplementary Table 2.

Point by point response to Reviewer 2 Comments

We are grateful for your comments which have helped us greatly improve our manuscript. As indicated in the responses that follow, we have taken your comments into account in our manuscript.

1*. However, the authors only look at high and low levels of infiltrating immune cells, control/normal tissue analysis is needed.*

Response: In this current study, we evaluated the infiltration of immune cells into the tumor microenvironment in patients with biliary tract cancer. Also, we investigated how well the infiltration of immune cells can predict clinical prognosis. Because healthy patients do not have tumor, it is impossible to evaluate immune cell infiltrations into their tumor microenvironment. In addition, it is difficult to obtain biliary tract specimens from healthy non-cancer-bearing patients.

*2.* *You may need to assess the co-staining of CD4+ and Foxp3 staining, since there may be overlap, to conclusively determine specific cell type, as well as CD3+ with CD8+/CD4+ cells (instead of them each individually).*

Response: I totally agree with your point. Performing double staining using various cell surface markers will allow us to understand immune cell infiltration in TME in more detail. However, our future goal is to examine how to apply this result to clinical practice, and we would like to replace it with a simple method. We evaluated CD3 positive cells as a pan-T cell marker. We found that CD3 is useful to understand the infiltration of immune cells in TME, although the accuracy decreased compared to each immune cell evaluation. In the future, we will investigate whether predicting prognosis is possible by evaluating immune cell infiltration with H & E staining. We added the paragraph in page 31-32, line 305-325. Also, we added Supplementary Figure 4 and Supplementary Table 2.

*3. Images in Figure 2 are hard to visualize. It may be necessary to use Immunofluorescence to visualize co-staining.*

Response: We added arrows to the stained immune cells in Figure 1. Also, we changed the contrast and magnification to improve the clarity of visualization.

*4.* *In the discussion, the authors state that role of CD8+ T cells as a cytotoxic role. This is not necessarily true. There are numerous subsets of CD8+ T cell and the authors did not access these.*

Response: We added the description of CD8 subsets in page 28, lines 264-267.
